# Fungal Drug Discovery for Chronic Disease: History, New Discoveries and New Approaches

**DOI:** 10.3390/biom13060986

**Published:** 2023-06-14

**Authors:** Thomas A. K. Prescott, Rowena Hill, Eduard Mas-Claret, Ester Gaya, Edie Burns

**Affiliations:** 1 Royal Botanic Gardens, Kew, Richmond TW9 3AB, Surrey, UK; 2 Earlham Institute, Norwich NR4 7UZ, Norfolk, UK

**Keywords:** fungi, drugs, self-resistance, ecological function, drug distribution, mechanism of action

## Abstract

Fungal-derived drugs include some of the most important medicines ever discovered, and have proved pivotal in treating chronic diseases. Not only have they saved millions of lives, but they have in some cases changed perceptions of what is medically possible. However, now the low-hanging fruit have been discovered it has become much harder to make the kind of discoveries that have characterised past eras of fungal drug discovery. This may be about to change with new commercial players entering the market aiming to apply novel genomic tools to streamline the discovery process. This review examines the discovery history of approved fungal-derived drugs, and those currently in clinical trials for chronic diseases. For key molecules, we discuss their possible ecological functions in nature and how this relates to their use in human medicine. We show how the conservation of drug receptors between fungi and humans means that metabolites intended to inhibit competitor fungi often interact with human drug receptors, sometimes with unintended benefits. We also plot the distribution of drugs, antimicrobial compounds and psychoactive mushrooms onto a fungal tree and compare their distribution to those of all fungal metabolites. Finally, we examine the phenomenon of self-resistance and how this can be used to help predict metabolite mechanism of action and aid the drug discovery process.

## 1. Introduction

**Figure 1:**
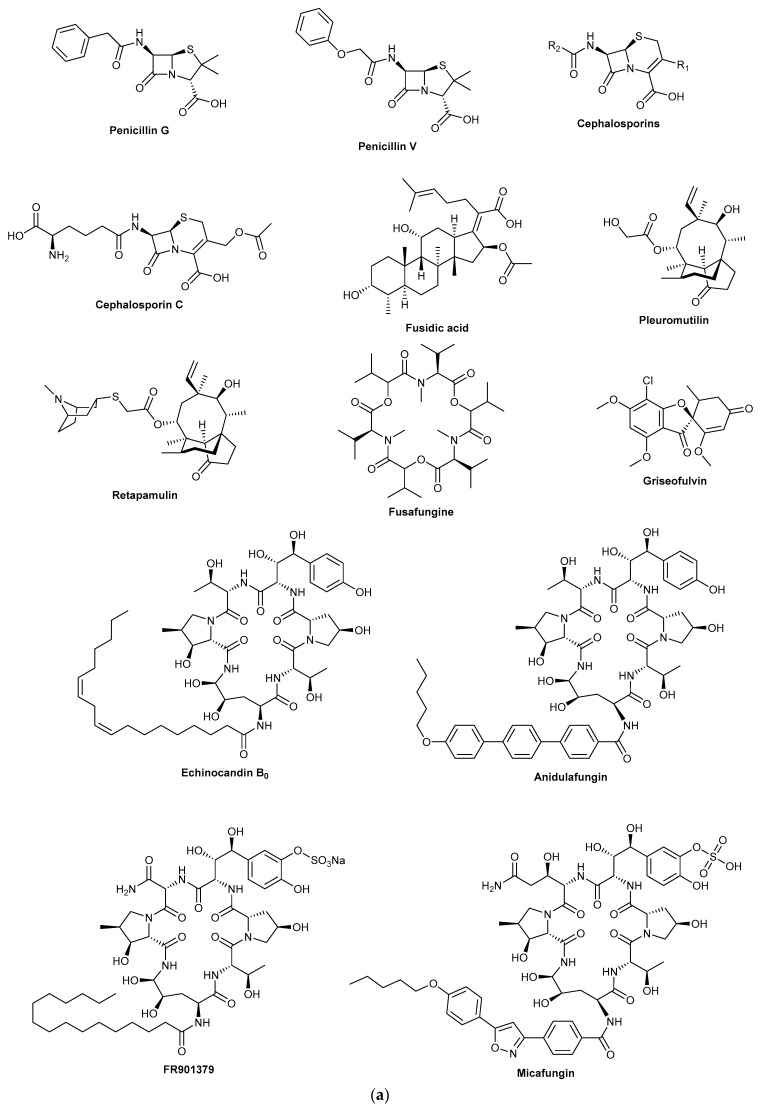

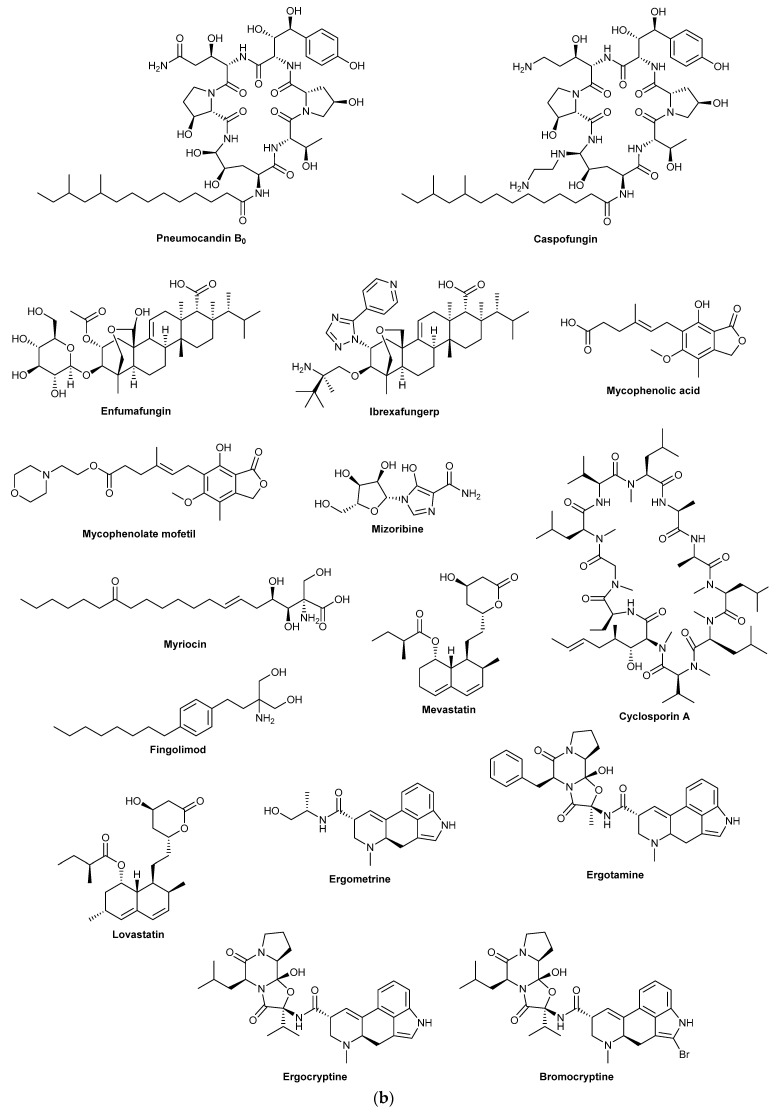
(**a**,**b**). Pharmaceutical drugs approved for human use that have a fungal origin.

Fungi are the source of some of the most important drugs ever discovered, including drugs of historic importance as well as recent blockbuster drugs. These compounds have found essential roles in treating chronic conditions, predominantly for chronic infections (antibiotics and antifungal drugs) autoimmune diseases (immunosuppressants) and hypercholesterolemia (the statins) (Table 1 and Figure 1). More recently, fungal-derived metabolites have been entering clinical trials to treat chronic diseases such as cancer and drug-resistant depression. In this review, we examine the discovery history of established fungal-derived drugs and examine new lead compounds that have recently entered clinical trials. Although different definitions for chronic disease exist, for this review we shall use the Centre for Disease Control and Prevention definition, in which chronic diseases are defined as conditions that last a year or more and require ongoing medical attention or limit activities of daily living or both [1].

The idea that fungi contain biologically active compounds is not new and pre-dates the rise of scientific methodologies. Indeed, various cultures have long made use of fungi as medicines and hallucinogens [2]. The isolation of pure chemical substances from fungi is not a recent endeavour either. Early examples of compound isolation date back to at least 1893 [3]. Despite this early knowledge of fungal bioactivity, the story of fungal drug discovery in many ways starts with the discovery of penicillin in the 1940s. Penicillin drew the attention of the scientific world to the incredible potential of fungi as a source of therapeutic small molecules. A chance observation of antibiotic antagonism by a contaminant mould on a Petri dish resulted in the eventual discovery of a highly effective antibiotic that would change the course of history.

The fungal contaminant on Alexander Fleming’s Petri dish was a species of *Penicillium* but how it got there is unclear. It may have drifted in the air from the laboratory of Charles La Touche, a mycologist working on *Penicillium* moulds whose laboratory was in the same building [4]. Alternatively, it may simply have come from the air in Fleming’s laboratory; spores of penicillin-producing fungi are after all highly ubiquitous in indoor environments. The fungal contaminant in question, which had previously been misidentified as *Penicillium chrysogenum* or *P. notatum* has recently been re-identified as *Penicillium rubens* [5]. Furthermore, the penicillin isolated from it at Oxford, UK was penicillin F (2-pentenylpenicillin), not penicillin G or penicillin V which are the penicillins used in medicine today [6]. Penicillin G (benzylpenicillin) was isolated in the United States using the strain *P. rubens* NRRL 1951, (the Wisconsin strain) which was found growing on a mouldy cantaloupe, in Peoria, Illinois. Regardless of its origin, penicillin was not simply a highly effective antibiotic; it was a medicine that changed perceptions as to what was possible in the treatment of bacterial infections. Penicillin is a beta-lactam antibiotic that inhibits the cross-linking of peptidoglycans which are a structural component of bacterial cell walls [7]. Since humans lack a cell wall, penicillin is able to kill bacteria without affecting human cells. Its discovery established fungi as an important source from which to look for therapeutic molecules and begged the question, what other useful medicines might be lurking in what would be later recognised as the kingdom Fungi?

The next major antibiotic discovery was cephalosporin C, which shares the same beta-lactam structure with the penicillins, and also shares a similar mechanism of action. The producing organism *Acremonium chrysogenum* had been isolated in 1945 from seawater close to a sewage outfall in Sardinia by local scientist Giuseppe Brotzu, who was investigating the epidemiology of typhoid [8,9]. Brotzu had noticed that although *Salmonella* was common in the sewers of Sardinia, bathers who swam in the sea rarely contracted *Salmonella* infections, and he reasoned there might be a process of “self-purification of seawater”. In trying to investigate this hypothesis, he came across a fungal organism that exhibited antibiotic antagonism on a Petri dish, and like Fleming before him, he reasoned it might have therapeutic value [9]. The fungal strain he isolated eventually made its way to Edward Abraham, who had worked on the isolation of penicillin in Oxford, and he was able to chemically isolate cephalosporin C in 1953. The discovery stimulated the development of the wider cephalosporin class of antibiotics which have proved effective against bacteria resistant to penicillin and are still in development today. An interesting parallel between the isolation of cephalosporin C and penicillin drugs is the length of time taken to chemically isolate the active metabolite due to the lack of advanced separation methods available at the time. Given the paucity of effective antibacterial medicines available, it may be pertinent to consider if isolation of a semi-pure fraction would have been sufficient to demonstrate the efficacy of these compounds as topical antibiotics. This could have been achieved in a very small fraction of the time taken to achieve the more complete isolation that took many years and significantly delayed the introduction of antibiotics.

Another valuable antibiotic from a similarly unpalatable source is fusidic acid. Fusidic acid, whose chemical isolation was described in 1962, was obtained from the fermentation of *Acremonium fusidioides* (reported at the time as *Fusidium coccineum*). This species was isolated from monkey faeces [10]. Unlike penicillin and cephalosporins, fusidic acid inhibits bacterial protein translation but manages to do so in a way that avoids inhibiting eukaryotic and therefore human protein translation. Fusidic acid is used topically to treat skin or eye infections [10].

A fungal-derived antibiotic that is infrequently mentioned is fusafungine, which is an antimicrobial and anti-inflammatory agent used for treating upper respiratory airway disease. It is a mixture of enniatins, obtained from *Fusarium lateritium*, and its anti-inflammatory mechanism of action is thought to involve the downregulation of expression of ICAM-1 and proinflammatory cytokines [11]. Fusafungine was first authorised in Europe in 1963 but was withdrawn in 2016 due to a risk of serious allergic reactions [12]. More recently, the antibiotic resistance crisis has focused researchers to re-examine neglected fungal antibiotics such as the pleuromutilins, originally isolated in the 1950s from an edible fungus *Clitopilus scyphoides*, (formerly *Pleurotus mutilus*) [13]. A synthetic derivative, retapamulin received approval in 2007 for the treatment of superficial skin infections caused by *Staphylococcus aureus* and *Streptococcus pyogenes.*

Paradoxically, fungi have also been an important source of antifungal drugs. Later on, we shall examine how fungi achieve self-resistance to the antifungal compounds they synthesise. An early example of an antifungal compound derived from a fungus is griseofulvin, first reported in 1938 from *Penicillium griseofulvum* [14]. Griseofulvin is used for treating dermatophyte infections of skin, nails and hair, and is believed to exert its antifungal activity by interfering with mitotic spindle microtubule function [15]. A more recent example of antifungal drugs derived from fungi is the echinocandins which are potent lipopeptide inhibitors of 1,3-βD-glucan synthase, an enzyme required for cell wall biosynthesis in fungi. The semisynthetic derivatives caspofungin, micafungin and anidulafungin are first-line antimycotics used for the treatment of invasive mycosis [16]. Caspofungin which received FDA approval in 2001 is a semisynthetic derivative of pneumocandin B_0_, isolated in 1992 from *Glarea lozoyensis*, which was discovered in water and sediment from a pond in a farm near Madrid [17,18]. Micafungin which gained regulatory approval in 2005 is a synthetic derivative of the hexapeptide FR901370 isolated from *Coleophoma empedra*, anidulafungin which gained approval in 2006 is a synthetic derivative of echinocandin B_0_ isolated from *Aspergillus spinulosprous* (formerly *Aspergillus nidulans* var. *echinulatus*) [16,18].

**Figure 2:**
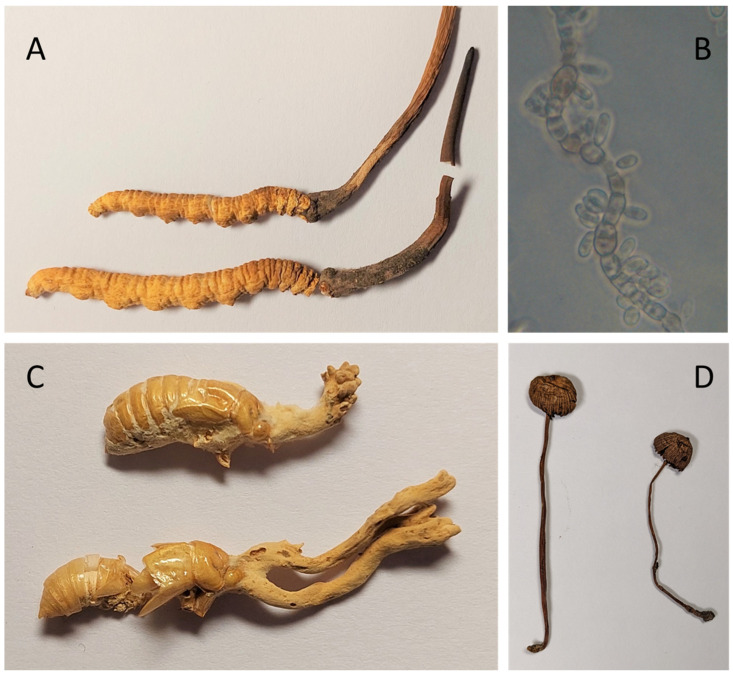
Recent fungal drug success stories. (**A**) *Ophiocordyceps sinensis* one of the sources of the compound cordycepin which has attracted significant attention from researchers leading to the investigative drug NUC-7738 for prostate cancer, photo credit to Lee Davies, RBG, Kew. (**B**) *Endoconidioma carpetanum* is an endophyte of juniper trees and source of the antifungal compound enfumafungin which led to the recently licenced antifungal drug ibrexafungerp. Photo credit Gerald F. Bills (**C**) *Isaria sinclairii*, emerging from its insect host. This species is one of the sources of the immunosuppressant compound myriocin which inspired the blockbuster multiple sclerosis drug fingolimod, photo credit to Lee Davies, RBG, Kew. (**D**) *Psilocybe mexicana is* the original source of the compound psilocybin which in 2018 was granted breakthrough therapy status by the Food and Drug Administration for treatment-resistant depression.

A very recent discovery is the triterpene glycoside enfumafungin which was isolated in the year 2000 from extracts of an endophytic species of *Hormonema* found to be living inside juniper trees [19]. Ibrexafungerp, a synthetic derivative, received approval in 2021 for the treatment of vulvovaginal candidiasis. The enfumafungin-producing fungal species has since been re-identified as *Endoconidioma carpetanum* Crous, comb. nov. MycoBank MB837886 (personal communication Gerald F. Bills), (Figure 2). Although these recent antifungal drug discoveries are all welcome additions to our antifungal drug armamentarium, the emergence of new fungal pathogens such as *Candida auris* means that antifungal drugs with a more diverse range of mechanisms of action will be needed to avoid the threat of drug resistance in the future [20].

Fungi have also been a highly successful source of immunosuppressant compounds. The first immunosuppressant drug to be discovered was mycophenolic acid in 1893, although its clinical use is much more recent. Mycophenolic acid was first isolated from *Penicillium brevicompactum* which is found in spoiled corn silage [3]. Mycophenolic acid inhibits inosine monophosphate dehydrogenase, thereby blocking guanine biosynthesis, which in turn blocks lymphocyte proliferation. It is used as an immunosuppressant for organ transplantation surgery [3]; a synthetic derivative mycophenolate mofetil was approved in 1995 for use during kidney transplantation [21]. A delayed-release tablet produced by Novartis and approved in 2004, achieved annual sales of USD 441 million in 2015 [21]. Mizoribine is an immunosuppressant compound isolated from *Penicillium dodgei* (formerly *Penicillium brefeldianum*) which was isolated from a soil sample collected from Hachijo Island in Japan in 1971. Like mycophenolic acid, mizoribine also inhibits inosine monophosphate dehydrogenase blocking lymphocyte proliferation in a similar manner [22]. It has been used in Japan since 1984 for the prevention of rejection in renal transplantation [22].

Notably, there are two fungal-derived immunosuppressant drugs that both come from entomopathogenic fungi. *Tolypocladium inflatum*, an entomopathogenic fungus whose spore forming structures emerge out of beetle larvae, is the source of cyclosporin A. Cyclosporin A inhibits the calcineurin pathway which blocks T-cell activation in humans and has proved pivotal for the field of organ transplantation [23]. Prior to the introduction of cyclosporin A, organ transplantation was considered more of an experimental field of surgery rather than a genuine therapeutic solution, with few patients surviving more than a few weeks [23]. Thus, like penicillin, cyclosporine is a medicine that changed perceptions of what is possible in human medicine. A more recent immunosuppressant success story is fingolimod, a treatment for multiple sclerosis that entered the market in 2011. Fingolimod’s structure took inspiration from the fungal metabolite myriocin, first discovered in 1972 from *Melanocarpus albomyces* (formerly *Myriococcum albomyces*), however, its immunosuppressant effects were not realised until 1994 when it was re-discovered from *Isaria sinclairii* (Figure 2) [24]. Fingolimod (the synthetic compound inspired by myriocin) is produced by Novartis and achieved blockbuster status with more than USD 1 billion in sales revenue in 2012, its second year of distribution [25].

Another therapeutic area where fungal-derived drugs have proved pivotal and highly lucrative is in the lowering of blood cholesterol levels. In the 1970s, Japanese scientist Akira Endo instigated a project to screen 6000 fungal extracts for inhibitors of cholesterol synthesis and in doing so discovered mevastatin (compactin) from *Penicillium citrinum*, a mould that infects *Citrus unshiu*, a type of Japanese orange [26]. Another statin drug, lovastatin was first reported from *Monascus ruber* in 1979 (reported as monacolin K) [27] and *Aspergillus terreus* in 1980 (reported as mevinolin) [28]. In 1987, lovastatin became the first statin to be granted FDA approval as a cholesterol-lowering drug, followed later on by mevastatin [29]. Statins act as competitive inhibitors of HMG-CoA reductase whose activity is required for cholesterol biosynthesis. Statins are one of the largest selling drug classes in the world, with sales in 2005 reported to be USD 25 billion [29].

Lastly, there are the ergot alkaloid drugs from *Claviceps purpurea* which grow on cereal crops resulting in their spoilage. Consumption of infected crops such as rye had been long known to be associated with ergotism, which causes burning sensations, convulsions and even hallucinations. The spores of *Claviceps purpurea* are carried by the wind and infect the ovaries of young rye plants. Their hyphae then take over the grain, replacing it with fungal tissue to form a hardened curved purple-coloured spur [30]. Ergot poisoning, which can cause paranoia and hallucinations occurs in people who ingest the infected grain. Outbreaks of ergotism, also known as St. Anthony’s Fire, are well documented in historical texts and were particularly prevelent in the Middle Ages in Europe with thousands of people affected [31]. Ergotamine, a constituent of *Claviceps purpurea* is a serotonin receptor agonist that was first isolated in 1918 [32]. Ergotamine tartrate has been used to treat migraines for many decades [33]. Ergot also has a long history of use in obstetric medicine, the first reference to the use of ergot in childbirth dates to Germany in 1582 [30]. Ergometrine maleate, a derivative of ergometrine, is used in the management of postpartum haemorrhage, it induces uterine contractions via agonist effects on myometrial 5-HT2 receptors [30,34]. Bromocriptine, a synthetic derivative of ergocryptine, is used for hyperprolactinaemia-related conditions in obstetric medicine. It is a dopamine agonist that blocks the release of prolactin from the pituitary gland [31,35].

Despite the very high revenues derived from certain fungal-derived drugs, by the late 1990s screening programs were failing to produce promising new compounds and were started to be wound down. The low-hanging fruit that produced useful compounds had all been discovered and it was becoming harder and more expensive to find promising drug leads from fungi. However, ninety-two years after the discovery of penicillin, fungal drug discovery is making a comeback. Several new fungal metabolites are currently in clinical trials and new commercial players are entering the field with the hope of applying novel genomics-based approaches to finding the next blockbuster drug.

## 2. Recent Fungal-Derived Drugs in Clinical Development

**Figure 3:**
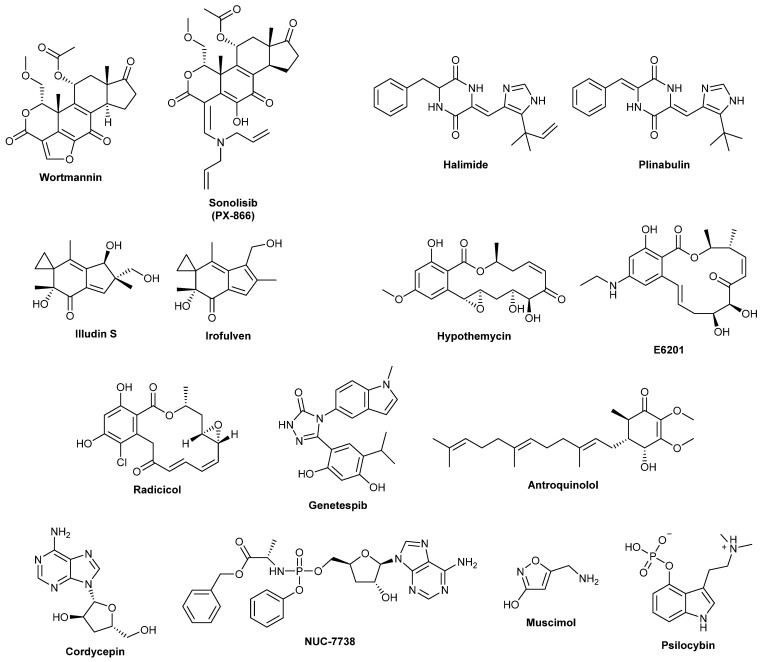
A selection of prominent compounds of fungal origin registered in the ChEMBL database (as of 2021) as having been entered into clinical trials (phases 1 to 4). Where relevant, the natural product parent and synthetic derivative are shown.

Fungal drug discovery is an ongoing process that constantly provides new drug leads, some of which enter clinical trials. In this section, we review recently developed drugs that have reached phase I clinical trials or greater (Table 2 and Figure 3). Although not all of these compounds will make it through to be approved drugs, their inclusion in clinical trials is indicative of excellent in vitro results.

Wortmannin is a furanosteroid metabolite from *Talaromyces wortmannii* (formerly *Penicillium wortmannii*) [36]. The original isolation of wortmannin dates back to 1957, although structure elucidation was completed later [37]. Although wortmannin has been known for years as a laboratory reagent for studying cell signalling in eukaryotic cells, interest in its development into a drug is more recent. Wortmannin is a potent inhibitor of phosphatidylinositol 3-kinase, which is an important drug target in a variety of different cancers [36]. PX-866, a synthetic derivative of wortmannin has entered phase II clinical trials for recurrent glioblastoma, the results of which were reported in 2017 [38]. Although the drug was relatively well tolerated, the overall response rate was low; furthermore, there was no statistically significant association between clinical outcome and the biomarkers selected for the study [38]. A phase II trial of PX-866 in patients with recurrent or metastatic castration-resistant prostate cancer reported in 2019, showed PX-866 had modest activity when used by itself [39].

Plinabulin is a synthetic derivative of two different fungal metabolites. Halimide, a metabolite first isolated in the late 1990s from *Aspergillus* sp. CNC-139 which was isolated from the Bahamian marine alga *Halimeda lacrimosa* and phenylahistin from *Emericella usta* (formerly *Aspergillus ustus*) [40]. Although originally characterised as a microtubule polymerisation inhibitor, it is now apparent that plinabulin can act as an immunomodulator to provide hematopoietic stem cell protection during chemotherapy [41]. Interestingly, plinabulin appears to act as an immunomodulator in bone marrow, increasing the number of peripheral progenitor stem cells. In a recent phase III trial reported in 2022, comparing plinabulin efficacy against the drug pegfilgrastim, plinabulin was found to have comparable efficacy to pegfilgrastim for the prevention of chemotherapy-induced neutropenia, with a better safety and immunosuppressive profile [41].

Irofulven is a DNA alkylating agent and synthetic derivative of illudin S which was first isolated in 1950 from cultures of *Omphalotus illudens* (formerly *Clitocybe illudens*) [42]. The same compound was then re-isolated from a related species by a research group that was apparently unaware of the previous isolation. To achieve this unintended re-isolation, they collected a metric ton of wild-collected *Omphalotus japonicus*, a poisonous bioluminescent mushroom which is often found growing on rotten beech trees in Japan and isolated 100 mg of pure illudin [43]. This chemical isolation strategy can clearly only be used for species that can be found growing in abundance in the wild. Irofulven, the drug developed from illudin S has reached phase II clinical trials for recurrent or persistent intermediately platinum-sensitive ovarian or primary peritoneal cancer. The results, reported in 2010, showed that although the drug was well tolerated by patients, it had modest effects in terms of clinical outcomes [44].

Hypothemycin is a resorcylic acid lactone first isolated in 1980 from *Hypomyces subiculosus* (formerly *Hypomyces trichothecoides*) [45]. E6201, a synthetic analogue of hypothemycin is a potent inhibitor of MEK kinase. Results from a 2018 phase I clinical trial show E6201 to be reasonably well-tolerated in patients with advanced solid tumours, with some evidence of clinical efficacy [46]. A potentially interesting outcome of the trial was that one patient who had malignant melanoma including brain metastases responded exceptionally well to the drug with overall survival extending beyond 8 years [47].

Radicicol is a resorcinol lactone which was first isolated in 1953 from the fungus *Monosporium bonorden* isolated from a soil sample from the Belgian Congo [48]. Radicicol is an inhibitor of HSP90, which is a molecular chaperone and an important drug target for certain types of cancer [49]. Ganetespib, a synthetic compound that took inspiration from radicicol was investigated in phase III clinical trials reported in 2020 [50]. The trial compared radicicol combined with docetaxel in advanced non-small-cell carcinoma, but the results did not demonstrate improved survival for patients [50].

Antroquinonol A is a ubiquinone derivative first isolated in 2007 from *Taiwanofungus camphoratus* (formerly *Antrodia camphorata*), a parasitic fungus native to Taiwan found growing on the inner heartwood of a decaying specimen of the tree *Cinnamomum micranthum* f. kanehirae (Hayata) S.S.Ying [51]. Antroquinonol is proposed to exert its effects by blocking Ras and Rho signalling through the inhibition of isoprenyltransferase. The drug was entered into phase II clinical trials for treating non-small-cell lung cancer [52]. The trial results reported in 2019, showed improved overall survival, however this was relative to historical data rather than a control. Notably, a separate research group that synthesised the compound from scratch were unable to replicate the previously published IC_50_ values [53]. Antroquinonol has recently been entered into a phase II trial to assess its efficacy in treating COVID-19 [54].

Cordycepin or 3′-deoxyadenosine is a nucleoside analogue first isolated in 1950 from the entomopathogenic fungus *Cordyceps militaris* but is also found in other *Cordyceps* spp. such as *Ophiocordyceps sinensis* (formerly *Cordyceps sinensis*) (see Figure 2) [55]. These species are endemic to the Himalayas at locations 2000 m above sea level and infect the larvae of *Thitarodes spp*. and *Lepidoptera* spp. moths which reside underground [56]. The fruiting bodies of the fungus, which grow out of the parasitised moth larvae to emerge above ground, are used as traditional medicines for a variety of conditions including cancer. The biological activity of cordycepin is attributed to its active metabolite cordycepin triphosphate which acts as a proapoptotic transcription inhibitor, although it has poor in vivo stability [57]. Cordycepin inhibits 3′ end processing of mRNAs and activates AMP-activated protein kinase [58]. Recently, NUC-7738, an improved analogue of cordycepin fused to a protective phosphoramidate cap, was developed and tested in a phase I clinical trial, the results of which were published in 2021. By collecting tumour samples from trial participants and examining them for changes in cancer biomarkers the authors were able to validate NUC-7738 as being both proapoptotic and inhibiting NFKB in vivo [57].

Muscimol, a psychoactive isoxazole first isolated from *Amanita pantherina* in the 1960s is a selective GABA_A_ receptor agonist [59,60]. A small-scale phase I clinical trial published in 2019 examined the use of convection-enhanced delivery of muscimol with the aim of creating a minimally invasive therapy for drug-resistant epilepsy [61]. Disappointingly, the trial was not able to confirm that muscimol effectively suppressed seizures using convection-enhanced delivery.

Although the examples of drugs in clinical trials given here are all pure substances, either chemically isolated from fungi or synthetic derivates of fungal compounds, there is also a long history of the use of fungi as medicines in traditional medicine and as hallucinogens. Guzman et al., 1998, reviewed the geographic distribution of mushrooms suspected of having hallucinogenic properties either by their traditional use or the presence of key metabolites and found that as many as 216 species may be hallucinogenic [2]. Mexico accounted for 76 species with 39% of these belonging to the genus *Psilocybe* [2]. In 1957 an amateur ethnomycologist Robert Gordon Wasson published an article in Life Magazine describing the strange visions he had experienced after taking *Psilocybe* mushrooms in Mexico. In the same year, psilocybin, the psychoactive fungal metabolite, was isolated by Albert Hoffman of Sandoz Laboratories from samples of *Psilocybe mexicana* collected in Mexico (see Figure 2) [62]. In order to perform bioactivity-guided fractionation of the active principle, Hoffman first carried out a paper chromatography separation of the chemical constituents, and then consumed each individual chromatographic fraction, assessing his own psychological state of mind as he proceeded. Using this unorthodox but effective procedure, Hoffman was able to identify the active component, which he then crystallised and named psilocybin [62]. To replicate this approach in the modern era would not be easy, as numerous ethical and safety issues would need to be resolved.

Nonetheless, his discovery proved important and there has recently been a resurgence of interest in psilocybin as a psychiatric medicine and a number of clinical trials have been carried out. In 2018, the U.S. Food and Drug Administration granted breakthrough therapy status for psilocybin for treatment-resistant depression [62]. A 2021 study examining the effects of psilocybin on major depressive disorder found psilocybin to be effective, with 13 participants (54%) all in remission at week 4 [63]. More recently, a clinical trial was carried out using single doses of 25 mg, 10 mg, or 1 mg psilocybin over 12 weeks in 233 patients with treatment-resistant depression [64]. The 1 mg dose was used in the control arm. Response to treatment was measured using the Montgomery–Åsberg depression rating scale which measures the severity of depressive episodes. The trial demonstrated a significant reduction in depression scores over a period of 3 weeks relative to the 1 mg dose. Although these data are impressive and act to further strengthen the evidence for psilocybin in treating depression, an important consideration is that it is difficult to blind the trials in a way that avoids participants knowing that they have received the active substance. This means that psychometric data may be biased by the patient’s realisation that they either have or have not been given the active substance. However, the use of the 1 mg control dose may help ameliorate this problem.

Another traditional fungal medicine that has attracted the attention of scientists is *Ganoderma lucidum*. This species has a long history of use in traditional Chinese medicine where it is referred to as Lingzhi, and in Japan where it is referred to as Reishi [65]. Various ancient texts refer to the medicinal properties of what is assumed to be *G. lucidum* which is purported to be useful in treating a wide variety of conditions, however, it is not clear that these traditional names can be unequivocally assigned to *G. lucidum*. Furthermore, the taxonomic situation within *Ganoderma* is not well resolved with the species and genus concepts confused. Similar fungi are found in *Fomes*, *Polyporus* and *Tomophagus* raising questions about the validity of some studies carried out on this species [65]. The generic type *G. lucidum* (Curtis) P. Karst., was apparently collected in Peckham, London, UK, but the holotype was subsequently lost [66]. Analysis of the ribosomal RNA sequence markers nuc-ITS and D2 of nuc-LSU indicate that specimens collected from Europe, America and South and East Asia do not represent a single species [66].

In vitro testing of constituents including ganoderic acids, 5, 6-dihydroergosterol and ergosterol peroxide showed modest effects on human cancer cells lines. Observed effects included induction of apoptosis and reactive oxygen species and inhibition of cell migration, but these occurred at concentrations somewhat below that which might be expected to explain any in vivo effects [67]. An alternative mechanism by which *G. lucidum* might exert its effects is via polysaccharides acting on immune cells such as B or T lymphocytes [68]. Interestingly, mice injected with breast cancer cells and treated with “Reishi” for 13 weeks showed a 50% reduction in tumour growth compared to control mice [69]. Several clinical trials have been carried out examining the effects of *G. lucidum* by itself or with chemotherapy agents in cancer patients. A meta-analysis was carried out on five studies including 373 people who were randomly selected to receive either *G. lucidum* or a placebo or other treatment [70]. The meta-analysis found that studies were of low to very low quality, due to lack of evidence as to how randomisation was achieved and low numbers of participants [70]. Furthermore, the studies did not measure survival after treatment. However, the authors did find that *G. lucidum* may improve the body’s T-cell immune response. Overall, the data indicated that *G. lucidum* showed better results when used in combination with other treatments [70].

Finally, aside from the drugs that have been discovered in fungi, there is also evidence to suggest that a mushroom-rich diet may help reduce the incidence of various types of cancer. This has been attributed to the presence of micronutrients present in certain species. A meta-analysis of the clinical trial data shows the evidence is strongest for breast cancer [71]. Various studies have pointed to the role of certain micronutrients present in mushrooms as likely candidates to explain the cancer-preventative properties suggested by clinical trials. Lentinan, a glucan molecule found in a variety of edible mushroom species including *Lentinus edodes* (Shitake mushrooms) [72], binds to cell surface receptors on monocytes and neutrophils, which in turn activates a variety of cell signalling pathways, which is suggested may enhance the effectiveness of the overall immune response towards cancer cells. Chemically purified lentinan has been approved for use as an adjuvant therapy for treating stomach cancer in Japan since 1985. Another compound that is suspected to play a role in cancer preventative effects of a mushroom-rich diet is ergothioneine [73]. Ergothioneine is rapidly taken up into cells via the organic cation transporter OCTN1, which is expressed in a variety of different human tissues. Once inside cells, it is proposed to act as an antioxidant thereby reducing cellular damage resulting from reactive oxygen species.

## 3. The Relationship between the Ecological Role of Fungal Metabolites in Nature and Their Therapeutic Use in Humans

An important consideration in fungal drug discovery is the ecological role of metabolites in natural ecosystems, and how this may be connected to their ability to act as useful agents in human medicine. A striking observation comes from the fact that of the clinically successful fungal-derived drugs, or their natural precursors, almost all possess some degree of antimicrobial activity. Obvious examples include antibiotics and antifungal metabolites such as penicillin, cephalosporins, griseofulvin, fusidic acid and echinocandins. Here, their role can easily be attributed to the competitive advantage gained by being able to dominate ecological niches by inhibiting competitor bacteria or fungi, and so gain preferential access to nutrients. Less obvious, however, is that metabolites such as cyclosporin A, lovastatin, mycophenolic acid and mizoribine are also potent antifungal compounds, even though their application in human medicine is in therapeutic areas, that are un-connected to antifungal use. Here, there is a less obvious but important connection between their antifungal activity which may provide an evolutionary advantage and their pharmacological mechanisms of actions in humans.

*A. terreus* for example, produces the antifungal compound lovastatin which inhibits the enzyme HMG-CoA reductase. In fungi, this enzyme is required for the synthesis of ergosterol, an important component of fungal membranes. It might be assumed that *A. terreus* gains a survival advantage when competing for nutrients in close proximity with lovastatin-susceptible fungal species [74]. However, in humans, HMG-CoA reductase is required for the synthesis of cholesterol. Thus, by opting to meditate its antifungal effects by inhibiting an enzyme that is conserved between fungi and humans, *A. terreus* has inadvertently produced a drug useful in treating high cholesterol levels in humans. In a similar vein, the fungal immunosuppressant compounds mycophenolic acid and mizoribine are both highly active antifungal compounds, and both compounds appear to exert their antifungal activity by inhibition of inosine monophosphate dehydrogenase, an enzyme required for purine synthesis in fungi. The same enzyme is also present in humans where purine synthesis is important for the proliferation of lymphocytes. Again, the choice of antifungal mechanism inadvertently provides a benefit in human medicine [75]. This crossover effect between the inhibition of an essential conserved fungal enzyme or receptor and the inhibition of a human homologue should not be surprising. A study in which 414 essential yeast genes were replaced with their human orthologs, found that nearly half of the yeast genes could be successfully replaced with the human version of the gene [76]. Thus, small molecule inhibitors of conserved fungal essential proteins are likely to also inhibit the human homologue, and occasionally this may give rise to unintended beneficial consequences.

The case of the immunosuppressant cyclosporin A shows a similar picture in terms of unintended outcomes for human pharmacology arising from ecological interaction in nature. Cyclosporin A is a cyclic peptide produced by the entamopathogenic fungus *T. inflatum* [23]. Cyclosporin A was originally discovered due to its antifungal properties but later found use as an immunosuppressant. In fungi, cyclosporin A is a selective inhibitor of the calcineurin pathway which controls the transcription of calcineurin-dependent genes [23]. Amongst the genes whose expression is controlled by the calcineurin pathway are those involved in virulence, hyphal growth and cell wall integrity [77]. Thus, cyclosporin A in all likelihood imparts a survival advantage to the fungal organism producing it when in direct close proximity and competition with cyclosporin A-susceptible fungal species. Yet, aside from its antifungal effects, cyclosporin A also appears to play a role in the immunosuppression of its insect host. *T. inflatum* is known for infecting beetle larvae and living inside them for a period of time before its spore-forming structures eventually emerge from its host. In higher eukaryotes such as insects and humans, calcineurin-dependent genes play an important role in immunity. Indeed, studies carried out in *Galleria mellonella* larvae treated with cyclosporin A demonstrated suppression of its humoral immune response [78]. Thus, it would appear that cyclosporin A enables *T. inflatum* to counter its host’s immune response during the infection process. This intriguingly mirrors its role in human medicine, where inhibition of the calcineurin pathway inhibits human T-cell activation resulting in immunosuppression. Indeed, the powerful immunosuppressive effects of cyclosporin A in humans proved pivotal in the development of the field of organ transplantation as discussed earlier. The ecological function of cyclosporin A may therefore in fact be two-fold. Firstly, to act as an immunosuppressant of the insect host of *T. inflatum* and once the insect host is parasitised, to prevent opportunistic fungal pathogens from forming a co-infection by virtue of its antifungal properties. Notably, *T. inflatum* also lives saprotrophically in soil, so cyclosporin A may provide a survival advantage over other soil-dwelling fungi due to its antifungal effects [23].

Cyclosporin A is not the only immunosuppressant derived from an entomopathogenic fungus. A relatively recent success in fungal drug discovery has been the development of the multiple sclerosis drug fingolimod, whose chemical structure took inspiration from the compound myriocin, produced by the entomopathogenic fungus *Isaria sinclarii* (see Figure 2) [24]. Like *T. inflatum*, *I. sinclarii* infects insect larvae; in the case of *I. sinclarii*, specifically the grubs of certain cicada species. *I. sinclarii* produces the compound myriocin which inhibits the biosynthesis of sphingolipids resulting in immunosuppression of the insect host, whereas the drug fingolimod acts on sphingosine receptors resulting in receptor internalisation, which prevents lymphocytes from exiting lymph nodes in the human patient [24]. This in turn, prevents autoimmune-mediated attack on myelin which is a hallmark of multiple sclerosis pathology. Although the mechanism of action of myriocin and fingolimod differ somewhat, it is interesting that both are able to exert immunosuppressive effects in their respective target organisms.

An obvious question that arises from the observation that so many fungal-derived drugs are themselves antifungal, is how does the producing fungal organism do this without killing itself? The answer appears to often lie with resistance genes that reduce the intracellular toxicity of the metabolites, thereby making their biosynthesis tolerable. Intriguingly, for compounds that are drug-like and interact with a specific receptor, such resistance genes are often a gene duplication of the actual pharmacological receptor of the compound. Cyclosporin A is one such example. A recent study aimed at identifying genes involved in cyclosporin A biosynthesis found that surprisingly, the biosynthetic gene cluster includes a copy of a gene similar to human cyclophilin A, the pharmacological target of cyclosporin A [79]. To investigate further, the authors created a deletion mutant in which the cyclophilin gene had been removed and then showed that this mutant was indeed sensitive to exogenous cyclosporin A whereas the wild-type strain was not [79].

Another example of the self-resistance phenomenon comes from the echinocandin-producing fungus *Pezicula radicicola*. Echinocandins noncompetitively inhibit the catalytic subunit of the enzyme β-1,3-glucan synthase, thereby inhibiting fungal cell wall synthesis. The authors found that adjacent to the echinocandin biosynthetic gene cluster was the gene FKS1 which encodes β-1,3-glucan synthase, the pharmacological target of the echinocandins [80]. The gene was found to be upregulated in the presence of exogenous echinocandin. Deletion of the gene rendered *P. radicicola* sensitive to exogenous echinocandins whereas the wild-type strain was resistant.

A further example comes from the immunosuppressant compound mycophenolic acid produced by *Penicillium brevicompactum*, which as discussed earlier exerts an antifungal activity by inhibition of the enzyme inosine-5′-monophosphate dehydrogenase. Analysis of the mycophenolic acid biosynthetic cluster revealed an extra copy of the inosine-5′-monophosphate dehydrogenase gene embedded in the cluster [81]. Heterologous expression of the gene in *Aspergillus nidulans* dramatically increased resistance to mycophenolic acid.

*A. terreus,* the producer of lovastatin, also makes use of self-resistance. Analysis of the lovastatin biosynthetic gene clusters shows the presence of a gene called *lvrA,* which is similar to HMG-CoA reductase, the pharmacological drug target of lovastatin and again, was shown to confer self-resistance [82]. A final example to illustrate self-resistance is fumagillin, a meroterpenoid from *Aspergillus fumigatus* that acts in humans as an angiogenesis inhibitor through the inhibition of human methionine aminopeptidase 2. Notably, the fumagillin biosynthetic cluster also encodes a fungal methionine aminopeptidase [83].

In each of these examples, although it is clear that self-resistance is being achieved through gene duplication of the pharmacological receptor of the antifungal metabolite, the precise mechanism through which self-resistance is achieved is less clear. Resistance could arise through increased expression of an enzyme or receptor compensating for enzyme or receptor inhibition, or a mutation in the enzyme or receptor that reduces receptor–ligand binding. However, it is also conceivable that the receptor acts as an inactivating binding protein during biosynthesis. The compound could then be transported out of the cell attached to the protein receptor using intracellular protein trafficking. Furthermore, with each of these examples, the compound in question is highly targeted in its mechanism of action. In the case of a promiscuous compound where there is no single defined receptor, this process would not provide a suitable means of achieving self-resistance.

## 4. Do Fungi Copy Plants to Produce the Same Drug-like Molecules?

Perhaps one of the more surprising twists in the story of drug-producing fungi is the strange phenomenon of certain fungal endophytes appearing to produce the same bioactive compounds as their plant hosts. An early example of this phenomenon was provided by *Ceriporiopsis andreanae* (formerly *Taxomyces andreanae*), a fungal endophyte of the Pacific yew tree *Taxus brevifolia* which is the source of the important chemotherapy agent paclitaxel. Evidence to support the production of paclitaxel by *C. andreanae* was provided by mass spectrometry of cultures, and incorporation of radiolabelled precursors into the fungus-produced paclitaxel [84]. The ecological function of this phenomenon is not clear but a recent study of another taxol-producing fungal endophyte *Paraconiothyrium* SSM001 provides some clues. In vitro, *Paraconiothyrium* SSM001 antagonised the growth of wood-decaying fungi including a species known to infect *T. brevifolia*, thereby supporting the idea that *Paraconiothyrium* SSM001 may protect its tree host from wood-decaying fungal pathogens. *Paraconiothyrium* SSM001 was shown to sequester taxol in intracellular hydrophobic bodies that were then released in the presence of wood-decaying fungal pathogens [85]. Intriguingly, *Paraconiothyrium* SSM001 was shown to migrate to potential pathogen entry points in its host tree and to release the taxol-containing bodies in a protective antifungal layer.

A key question that needs to be resolved here is how did the plant and the endophyte evolve to produce the same metabolite? One explanation appears to be horizontal gene transfer from the plant to the fungal endophyte, but firm evidence to support this hypothesis is currently lacking. Indeed, some authors have questioned whether *Ceriporiopsis andreanae* is indeed producing paclitaxel *de novo*. A carefully conducted study that examined various fungal endophytes reported to be capable of taxane biosynthesis found that although the compounds could be detected at trace amounts using LC-MS there was no evidence for the necessary biosynthetic gene clusters that would allow their biosynthesis [86]. Instead, they propose that the detection of taxanes can be best explained by the accumulation of the compounds from the host tree, and subsequent gradual release into the culture medium. They suggest the non-polar taxoid molecules are sequestered in lipophilic cell structures of the fungal endophytes. Recently, a comparative genomics assessment of *Taxomyces andreanae* demonstrated that rather than belonging to the *Ascomycota* it is, in fact, a *Basidiomycete* [87]. The authors point out that it is almost inconceivable that a molecule like taxol could have arisen convergently in the *Ascomycota*, *Basidiomycota* and plants.

Paclitaxel is not the only example where this phenomenon has been observed. For example, trace quantities of the vinca alkaloids, vincristine and vinblastine were detected from axenic cultures of *Fusarium oxysporum*, an endophyte of *Catharanthus roseus*, famous as the source of the vinca alkaloid leukaemia drugs [88]. Similarly, camptothecin is a cytotoxic topoisomerase I inhibitor, produced by the tree *Camptotheca accuminata* and synthetic derivatives of camptothecin such as topotecan are used as cancer chemotherapy drugs. Trace amounts of camptothecin have been detected in the cultures of a strain of *Fusarium solani*, a fungal endophyte isolated from the tree, however after three generations of growth the levels of camptothecin had dropped considerably. In this instance rather than the fungal endophyte producing camptothecin de novo, the authors propose a mechanism in which the endophyte produces camptothecin precursors that are then used by the host plant to produce camptothecin [89]. *F. solani* is known to be able to absorb polyaromatic compounds from cell culture media and store them within intracellular compartments, [86]. If it is able to absorb compounds from the culture medium and then store them intracellularly it is also possible that *F. solani* absorbs camptothecin from its host tree, and then slowly releases the compounds into the culture medium.

Given the uncertainty surrounding the origin of the compounds in these studies, it is important that future investigations of this phenomenon make use of fungal culture media containing radiolabelled precursors. Incorporation of the radiolabelled precursor into the metabolite in question then constitutes stronger evidence for biosynthesis by the fungal endophyte as opposed to accumulation and release. Furthermore, to provide unequivocal evidence of metabolite biosynthesis by the endophyte in question, the metabolite should be chemically isolated in a quantity that exceeds the mass of endophyte inoculum used to inoculate the culture medium. Useful supporting evidence would also be to identify putative biosynthetic gene clusters in the genome of the suspected producing organism. Interestingly, one example where biosynthetic gene clusters have been identified is the case of *Fusarium* species that produce the gibberellin plant hormones [90]. Analysis of the biosynthetic gene clusters involved shows differences between plants and fungi, suggesting that rather than horizontal gene transfer, the biosynthesis has evolved by convergent evolution [91].

Even if the detection of certain compounds in endophyte cultures represents accumulation rather than biosynthesis, the phenomenon is still interesting and raises questions as to its ecological function. One intriguing possibility is that by accumulating the same cytotoxic agent as its plant host, the fungal endophyte can deliver an antifungal compound to specific locations in the plant using a compound that the host plant is already immune to. For example, in the case of *C. accuminata*, it has been shown that three amino acid substitutions in the plant’s topoisomerase I gene provide protection from camptothecin [92]. Thus, by being able to move within the host plant to the site of fungal infection it is possible that fungal endophytes are able to deliver the antifungal compounds at higher concentrations where they are needed without harming the host plant.

## 5. Fungal Self-Resistance Mechanisms Provide Useful Information for a New Wave of Fungal Drug Discovery

The commercial imperative to discover novel clinically effective therapeutics from fungi has driven drug discovery programs based on large fungal strain collections. In this conventional model of fungal drug discovery, environmental samples such as soil, water or plant material are collected, and then individual fungi are isolated and genotyped. Individual fungal isolates can then be grown on different culture media to stimulate secondary metabolite production. Extracts of fungal mycelium or fungal culture broths can then be extracted and assayed for biological activities of interest, using LC-MS to avoid re-isolation of known metabolites. It is this approach that is behind the discovery of ground-breaking drugs such as cyclosporin A and statins.

For example, at Novartis, (one of the last remaining large pharmaceutical companies involved with fungal natural products), there is a fungal culture collection compromising tens of thousands of strains sourced from partners all over the world, a collection that is still growing year by year. Fungal cultures that produce extracts with the desired biological activity are re-cultured, sometimes at a very large scale, using tens of thousands of litres of culture medium which are then extracted with similar volumes of organic solvents. After chemical purification, this often yields only a few milligrams of pure isolated compound. Although this approach is behind the discovery of highly important drugs, there are nonetheless drawbacks. For example, some fungal species are very slow growing or cannot be cultured at all. Additionally, many fungi possess cryptic biosynthetic gene clusters that are not expressed during axenic culture. Evidence for this comes from the discrepancy between the number of biosynthetic gene clusters within a given fungal species and the number of secondary metabolites actually detected [93]. Such genes may only be expressed when mycelia come into contact with a host plant or other fungal species. Therefore, there may be many novel bioactive metabolites that are missed using standard culture methods.

However, new commercial players have recently emerged that aim to streamline the fungal drug discovery process. Hexagon Bio is one such company. Their approach is based on two separate innovations. Firstly, by creating a carefully curated set of promoter sequences in *Saccharomyces cerevisiae*, each of which is mutually non-overlapping, they are able to heterologously express cryptic biosynthetic genes, thereby side-stepping the difficult and time-consuming process of optimising culture conditions for the production of a given secondary metabolite [94]. Secondly, by making use of the self-resistance phenomena discussed earlier, they are able to rapidly identify biosynthetic gene clusters and even make an informed guess to the likely mechanism of action of the encoded metabolite before they have carried out chemical isolation of the active compound. For example, if in a blind screening process, they were to encounter *A. terreus* (the producer of lovastatin), without any foreknowledge of what metabolites *A. terreus* synthesises, they would be able to surmise that the fungal organism in question may produce a compound acting on fatty acid synthesis. This is because a protein resembling the pharmacological target of lovastatin, (HMG-CoA reductase) is duplicated in the biosynthetic gene cluster to provide self-resistance.

This provides an efficiency gain in the drug discovery process; a likely mechanism of action can be assigned to a fungal metabolite before even knowing what the fungal metabolite is. As HMG-CoA reductase is a clinically important drug target with a human homologue, they can next decide to clone the associated biosynthetic genes into yeast using their proprietary set of promoters, and then identify the compound in question. By comparison, using the conventional drug discovery approach, *A. terreus* would need to be cultured at scale using a suitable culture medium to stimulate lovastatin production before chemical isolation could be carried out. Next, the active compound (lovastatin) would need to be subjected to a suitable assay to elucidate its mode of action. A yeast chemical genetic assay would be a sensible choice. This assay measures the sensitivity of a genome-wide yeast deletion library to the test compound in question. From the library of deletion strains, the hypersensitivity of an HMG-CoA reductase deletion strain would provide evidence to support this mechanism of action. Thus, the presence of the duplicated HMG-CoA reductase gene provides an elegant means of side-stepping the experimental bottleneck associated with the conventional fungal drug discovery approach.

An additional benefit of looking for self-resistance genes to understand the compound mechanism of action is that only compounds that have selectivity for a specific receptor will use gene duplication to provide self-resistance. Promiscuous compounds that lack a targeted mode of action, for example, non-specific DNA binding or membrane perturbation, will not be able to achieve self-resistance in this manner since they lack a specific protein receptor. Compounds acting through these mechanisms such as these are sometimes referred to as junk compounds and make up a large proportion of “bioactive” natural products reported in the literature. Being able to eliminate them and only focus on more targeted compounds provides a clear advantage. An important caveat, however, is that fungal organisms producing compounds that act on a human pharmacological target that does not have a fungal homologue would not be identified using this approach. Therefore, there may be fungal compounds that act on human receptors that do not have fungal homologues and these need to be isolated and tested using a conventional approach, or they will be missed. In reality, both approaches may be needed to maximise the chances of finding the next blockbuster drug.

## 6. Distribution of Bioactive Compounds in the Fungal Kingdom: Where to Search for the Next Blockbuster Drug

One question that is yet to be answered for fungi concerns the distribution of bioactive compounds in the kingdom: are they distributed randomly or are certain clades particularly capable of synthesising such compounds? To obtain an initial glimpse into this complex question we extracted and synthesised available information on fungal-derived drugs and bioactive compounds. We chose four different bioactivity categories. Approved drugs, drug precursors, or lead compounds of fungi present in clinical trials phase 0 to 4 in the ChEMBL database as of 2021. Approved drugs or compounds that have been entered into clinical trials are likely to be potent and selective inhibitors of a given pharmacological target or would not have been chosen as lead compounds or achieved success in a given therapeutic area. For the producing organisms to produce such compounds requires either considerable luck or alternatively the honing through evolutionary processes of the ligand structure such that it docks with high affinity to its cognate receptor. We also include antibacterial and antifungal compounds reported in the Journal of Antibiotics between the years 1961 to 2003. Such compounds are not necessarily potent and selective, such as lead drug compounds but do nonetheless possess demonstrable biological activity. For comparison, we also plot the distribution of all secondary metabolites reported from fungi in the literature up to 2020, taken from the Natural Product Atlas database. This provides a useful reference to indicate natural biases for where natural product researchers choose to look for novel bioactive compounds in the fungal kingdom. Finally, we also include fungi believed to be psychoactive based on their traditional use or the presence of key metabolites, using data from Guzman et al. (Guzman, 1998).

**Figure 4:**
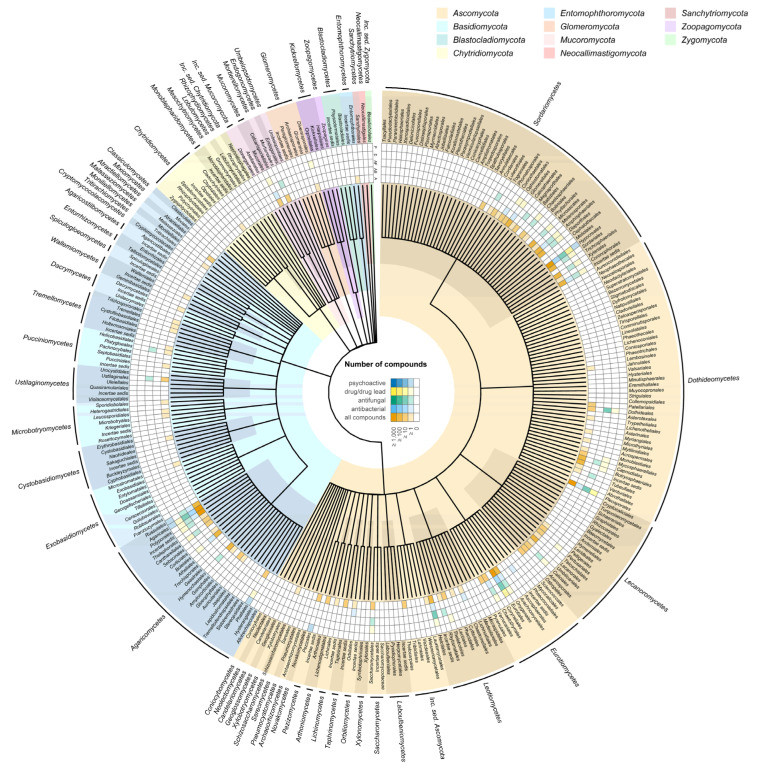
Distribution of fungal orders across the fungal kingdom that have been reported to produce different categories of bioactive compounds, each represented in fungal orders. The data indicate that either research efforts are biased towards certain orders or that certain orders are particularly capable of producing bioactive compounds. (Dark blue) fungi that are believed to be psychoactive based on their traditional use or the presence of key metabolites, taken from Guzman et al. [2]. (Yellow) fungi that are recorded as the source of approved drugs, drug precursors or lead compounds in clinical trials phase 0 to 4 in the ChEMBL database as of 2021. (Green) antifungal compounds reported in the Journal of Antibiotics between the years 1961 to 2003. (Light blue) antibacterial compounds reported in the Journal of Antibiotics between the years 1961 to 2003. (Orange) all fungal secondary metabolites reported in the literature in 2020, taken from the Natural Product Atlas database.

Using a taxonomic backbone tree, we mapped compound distribution at the order level showing all fungal classes (Figure 4). The tree does not depict the phylogenetic relationships amongst the fungal groups, so compound distribution does not mirror the phylogenetic relatedness of taxa, but it gives us a preliminary insight into which fungal groups may be more prolific producers of bioactive compounds and/or which taxa have received more attention from investigators. We observe a stark bias towards certain taxonomic groups. This is not a surprise, and it is due to a combination of factors. Fungal drug discovery has been historically focused on taxa such as *Penicillium* and *Aspergillus* that can be easily cultured in vitro whilst larger specious-rich and ubiquitous groups including those that spoil food or produce mushrooms are also more easily targeted by investigators. Additionally, those fungi with a wider range of lifestyles, especially those that establish symbiotic interactions with organisms from other kingdoms, seem to be more capable of producing bioactive compounds. However, even with the recent surge of fungal genomic data, uncertainly as to where certain taxa should be placed within the fungal tree of life makes it difficult to make definitive predictions as to which clades may prove fruitful for natural product drug discovery. Nonetheless, here we summarise the broad trends revealed in Figure 4.

The largest phylum *Ascomycota* is also the one that contains the highest number of orders containing bioactive species. The large class of *Sordariomycetes* stands out as the one having by far the most orders with approved drugs, and/or antifungal and antibacterial activity. Within this class, the order *Hypocreales*, with 2647 described species, encompasses mostly systemic endophytes, plant pathogens and fungal and insect parasites. The genus *Acremonium* (family *incertae sedis*), contains saprotrophs and opportunistic animal pathogens. The families *Ophiocordycipitaceae* and *Cordycipitaceae*, also in the Hypocreales, are especially well known for their medical and culinary value, a notable example being *Tolypocladium inflatum*. The genus *Trichoderma* (*Hypocreaceae*), present in most soils and generally observed to be easily culturable has also yielded numerous bioactive metabolites. Finally, the family *Clavicipetaceae*, which contains many taxa known to produce alkaloids toxic to animals is also found in the *Hypocreales*. Interestingly, the closest relatives to this order do not appear to be such prolific producers of bioactive compounds. Whereas the order *Glomerelalles* contains drugs and antibacterial compounds, and *Microascales* antifungal compounds, the closest *Coronophorales*, *Falcocladiales* and *Torpedosporales*, albeit much smaller groups, lack any records of compound activity in our datasets. One of the main differences for these rarer groups is that being mostly saprotrophs, they have a narrower nutritional range.

Apart from *Hypocreales*, other orders in the *Sordariomycetes* that contain significant numbers of bioactive compounds are the *Chaetosphaeriales* which are mostly endophytes, the *Amphisphaeriales* and *Diaporthales* which include plant pathogens, the *Ophiostomatales* known to be insect parasites, the *Sordariales*, a saprotroph order and the *Xylariales*, one of the most prolific lineages of secondary metabolite producers with many endophytic species. Except for the closely related *Amphisphaeriales* and *Xylariales*, the other orders associated with bioactivity are spread across the tree, often sister to groups that apparently lack bioactivity. It is important to note that several bioactive compounds are reported from species with an *incertae sedis* placement in the *Sordariomycetes* and may not be assigned to a particular family or order.

In the next class, the *Dothideomycetes*, the *Pleosporales*, produces a relatively higher number of drugs, and the species contained within it exhibit a broad range of lifestyles, being saprotrophs, parasites, pathogens, epiphytes and endophytes. Five other orders present drug lead and/or antifungal, but no antibacterial activity. One of them, the *Capnodiales*, is the second largest order and encompasses a diversity of lifestyles including saprotrophs, plant and human pathogens, mycoparasites, rock-inhabiting fungi, lichenised, epi-, ecto- and endophytes. The *Capnodiales* are closely related to the *Dothideales*, an order of mostly plant pathogens with a high number of antifungal compounds. The *Botryosphaeriales*, which contain drug-producing species also present a diverse ecology, being saprobic, endophytic, plant pathogenic and opportunistic human pathogens.

In the *Eurotiomycetes*, the *Eurotiales*, *Onygenales* and *Chaetothyriales* are unsurprisingly prolific producers of bioactive compounds. The genera *Aspergillus* and *Penicillium* belong to the *Eurotiales* along with other economically important groups. For *Aspergillus* and *Penicillium* a key question is to what extent their success in the field of natural product drug discovery can be attributed to their biosynthetic capabilities or to their popularity with researchers due to being easy to culture. A combination of both would appear to be likely. Like other bioactive-rich groups discussed here, the *Eurotiales* present a range of ecological roles, ranging from being saprotrophs to human pathogens.

Compared to the *Ascomycota*, the phylum *Basidiomycota* presents a much more concentrated distribution of known drugs and bioactive compounds many of which are found in the class *Agaricomycetes*. This is not a surprise, as this class contains the classic mushroom-forming fungi, including some of the most visible and best-known groups including numerous edible species and taxa associated with traditional medicine and psychoactivity in various cultures. The orders *Agaricales* and *Russulales* contain the highest number of bioactive compounds. Compared to the *Ascomycota*, the nutritional modes in this class are much more focused, with most species being either saprophytes or mycorrhizal symbionts or both. Finally, amongst the smaller and more early diverging groups, the *Mucoromycetes* contain saprotrophic species and some parasites or pathogens of animals and plants.

## 7. Conclusions

In this review, we have shown that despite the importance of fungal-derived drugs to the treatment of chronic diseases, in many cases their discovery is purely serendipitous or the result of random screening. Future fungal drug discovery programs need to consider the ecological role metabolites may have in nature and use this to provide more intelligent screening methods. Furthermore, finding more efficient discovery methods that avoid the inherent bottlenecks in the fungal drug discovery process is key to unlocking the medicinal potential of the fungal kingdom. The inerrant disparity between the chemical diversity of fungi suggested by genomic analysis of biosynthetic gene clusters and the actual number of compounds discovered highlights the need for innovative new methods, but progress is now being made. Recent advances in heterologous expression of fungal biosynthetic gene clusters in yeast are making compound isolation and discovery more efficient and overcoming some of the experimental bottlenecks that have been a hallmark of fungal drug discovery in the past. An understanding of the importance of metabolite self-resistance is stimulating a renaissance in pharmaceutical bioprospecting from fungi. The ability to gain clues to the mechanism of action of compounds produced by a given species before any chemical analysis has even taken place is proving to be a powerful investigational tool, and has attracted significant investment from the commercial sector. These advances may yet see new fungal-derived drugs reaching the clinic in years to come. Even without these new experimental tools, research into previously known bioactive fungal metabolites has led to a range of fungal small molecules being tested in clinical trials, and a small number have recently received approval for use in humans.

Future fungal drug discovery programs may benefit from a big picture view that takes into account patterns in the distribution of bioactive metabolites across the fungal kingdom, but this may involve examining traits such as fungal lifestyle and nutritional characteristics rather than simple taxonomic relationships. Our own analysis clearly proves the difficulty in revealing obvious patterns that may aid drug discovery predictions. Taxonomic relatedness does not seem to be a good predictor, at least with the bioactivity data sets used here. However, lifestyle flexibility may be a good indicator of bioactive metabolite production in the *Ascomycota*, but not in other groups. The fact that the fungal tree of life has so many unknowns and that so many bioactive species still have not been correctly placed, suggests that we may not yet be ready to use this type of information to guide drug discovery. Improved resolution of fungal genomic data combined with more densely sampled phylogenomic trees may help improve the situation. Additionally, a certain level of boldness is required; searching in previously unexplored groups may lead to the discovery of novel metabolites with novel mechanisms of action.

## Figures and Tables

**Table 1:** Approved drugs of fungal origin, including their natural product parent molecule and where relevant their synthetic derivates. Notes on their use and mechanism of action are also given. Note fusafungine has now been withdrawn from use in many jurisdictions. Additionally, note, although the design of fingolimod took inspiration from the structure of myriocin they have different mechanisms of action.

Natural Product	Synthetic Derivative	Original Source Organism	Use	Mechanism of Action
Penicillin G	-	*Penicillium rubens*	Antibiotic	Inhibition bacterial cell wall peptidoglycan synthesis
Penicillin V	-	*Penicillium rubens*	Antibiotic	Inhibition bacterial cell wall peptidoglycan synthesis
Cephalosporine C	Cephalosporins	*Acremonium chrysogenum*	Antibiotic	Inhibition bacterial cell wall peptidoglycan synthesis
Fusidic acid	-	*Acremonium fusidioides*	Antibiotic	Inhibition of bacterial protein synthesis, prevents translocation of elongation factor G
Pleuromutilin	Retapamulin	*Clitophilus scyphoides*	Antibiotic	Inhibition of bacterial protein synthesis. Binds to 50S ribosome subunit and inhibits peptidyl transferase centre
Fusafungine	-	*Fusarium lateritium*	Antimicrobial and anti-inflammatory	Inhibition of cytokine expression from alveolar macrophages by various mechanisms
Griseofulvin	-	*Penicillium griseofulvum*	Antifungal drug	Interferes with fungal microtubule polymerisation
Echinocandin B_0_	Anidulafungin	*Aspergillus spinulosprous*	Antifungal drug	Inhibits fungal cell wall synthesis by inhibiting glucan synthase
FR901379	Micafungin	*Coleophoma cylindrospora*	Antifungal drug	Inhibits fungal cell wall synthesis by inhibiting glucan synthase
Pneumocandin B_0_	Caspofungin	*Glarea lozoyensis*	Antifungal drug	Inhibits fungal cell wall synthesis by inhibiting glucan synthase
Enfumafungin	Ibrexafungerp	*Endoconidioma carpetanum*	Antifungal drug	Inhibits fungal cell wall synthesis by inhibiting glucan synthase
Mycophenolic acid	Mycophenolate mofetil	*Penicillium* *brevicompactum*	Immunosuppressant	Inhibition of lymphocyte proliferation (inosine-5′-monophosphate dehydrogenase inhibition)
Mizoribine	-	*Penicillium brefeldianum*	Immunosuppressant	Inhibition of lymphocyte proliferation (inosine-5′-monophosphate dehydrogenase inhibition)
Cyclosporin A	-	*Tolypocladium inflatum*	Immunosuppressant	Inhibition of T lymphocyte proliferation (binds to cyclophilin A inducing inhibition of calcineurin)
Myriocin	Fingolimod	*Melanocarpus albomyces*	Immunosuppressant, (multiple sclerosis)	Myriocin inhibits of sphingolipid synthesis. Fingolimod is a sphingosine 1-phosphate receptor agonist
Mevastatin	-	*Penicillum citrinum*	Cholesterol lowering	Inhibits cholesterol synthesis by inhibition of HMG-CoA reductase
Lovastatin	-	*Monascus ruber, Aspergillus terreus*	Cholesterol lowering	Inhibits cholesterol synthesis by inhibition of HMG-CoA reductase
Ergotamine	Ergotamine tartrate	*Claviceps purpurea*	Antimigraine agent	Cerebral vasoconstriction (alpha-adrenergic blocker)
Ergometrine	Ergometrine maleate and other compounds	*Claviceps purpurea*	Management of postpartum haemorrhage	Induces uterine contraction via agonist effects on myometrial 5-HT2 receptors
Ergocryptine	Bromocriptine	*Claviceps purpurea*	Hyperprolactinaemia related conditions in obstetric medicine	Dopamine agonist blocks the release of prolactin from the pituitary gland
Lentinan	-	*Lentinus edodes*	Adjuvant for cancer chemotherapy	Immunomodulator, binds to and actives various extracellular receptors in monocytes and neutrophils

**Table 2:** Compounds of fungal origin, including their natural product parent molecule and semisynthetic derivates, currently in clinical trials at phase I or greater.

Compound	Synthetic Derivative	Original Source Organism	Therapeutic Area	Mechanism of Action
Wortmannin	PX-866	*Talaromyces wortmannii*	PX-866 for metastatic castration-resistant prostate cancer, (2019 phase II clinical trial).	Phosphatidylinositol 3-kinase inhibitor
Halimide	Plinabulin	*Aspergillus* sp. CNC-139 *Aspergillus ustus*	Plinabulin for prevention of docetaxel-induced neutropenia during cancer treatment (2022 phase III clinical trial).	Plinabulin is a microtubule polymerisation inhibitor and separately stimulates T-cell activation
Illudin S	Irofulven	*Omphalotus illudens*	Irofulven for recurrent or persistent intermediately platinum-sensitive ovarian or primary peritoneal cancer (2010 phase II trial).	Irofulven is a DNA alkylating agent
Hypothemycin	E6201	*Hypomyces trichothecoides*	E6201 for advanced solid tumours, expanded to advanced melanoma (2018 phase I trial).	E6201 is an ATP-competitive MEK1 kinase inhibitor
Radicicol	Ganetespib	*Monosporium bonorden*	Ganetespib with docetaxel for advanced non-small-cell lung cancer (2019 phase III).	Ganetespib is an inhibitor of the molecular chaperone HSP90
Antroquinonol	-	*Taiwanofungus camphoratus*	Cancer, (non-small cell lung cancer) (2014 phase II).	Inhibits Ras and Rho signalling through inhibition of isoprenyltransferase
Cordycepin	NUC-7738	*Cordyceps militaris*	NUC-7738 for patients with advanced solid tumours or lymphoma (2021 phase I trial).	Cordycepin is a nucleoside analogue, inhibits transcription, inhibits 3′ end processing of mRNAs, activates AMP-activated protein kinase. NUC-7738 shown to be pro-apoptotic and NFKB inhibitor in human patients
Muscimol	-	*Amanita pantherina*	Drug-resistant epilepsy (2019 phase I).	Receptor agonist for GABA_A_-R subtypes
Psilocybin	-	*Psilocybe mexicana*	In 2018 U.S. Food and Drug administration granted breakthrough therapy status for treatment-resistant depression.	Psilocybin binds with high affinity to the 5-HT_2A_ serotonergic receptor subtype

## Data Availability

Not applicable.
